# The “Brain's Traffic Map” Reveals Neural Pathways Linked to Coronary Microvascular Dysfunction in Women

**DOI:** 10.1002/brb3.71559

**Published:** 2026-06-25

**Authors:** Arzu C. Has Silemek, Jeffrey C. Wertheimer, Janet Wei, Yibin Xie, Mitzi Gonzales, Debiao Li, Oana Dumitrascu, Sarah Kremen, Zaldy S. Tan, Micheal D. Nelson, C. Noel Bairey Merz, Pascal Sati, Wei Gao

**Affiliations:** ^1^ Department of Neurology Cedars‐Sinai Medical Center Los Angeles California USA; ^2^ Department of Biomedical Sciences and Imaging Biomedical Imaging Research Institute (BIRI) Cedars‐Sinai Medical Center Los Angeles California USA; ^3^ Department of Physical Medicine and Rehabilitation Cedars‐Sinai Medical Center Los Angeles California USA; ^4^ Barbra Streisand Women's Heart Center Smidt Heart Institute Cedars‐Sinai Medical Center Los Angeles California USA; ^5^ Department of Neurology Mayo Clinic College of Medicine and Science Scottsdale Arizona USA; ^6^ Departments of Neurology and Medicine Cedars‐Sinai Medical Center Los Angeles California USA; ^7^ Department of Kinesiology, College of Nursing and Health Innovation The University of Texas at Arlington Arlington Texas USA

**Keywords:** brain–heart axis, cognition, coronary microvascular dysfunction, neuroimaging, Unified Structural and Functional Connectivity, women's cardiovascular health

## Abstract

**Background:**

The brain–heart axis is central to vascular health, yet no imaging biomarkers capture integrated dysfunction across neural and coronary microvascular networks. Although coronary microvascular dysfunction links to cognitive decline, neural correlates connecting cerebral efficiency with coronary physiology remain unclear.

**Objectives:**

To determine whether the Unified Structural and Functional Connectivity (USFC)—a multimodal magnetic resonance imaging (MRI) “traffic map” of brain network efficiency—predicts coronary endothelial function and cognition in women with ischemia and no obstructive coronary artery disease (INOCA).

**Methods:**

Thirty‐three women with suspected INOCA from the Women's Ischemia Syndrome Evaluation (WISE) study (NCT03876223) underwent invasive coronary function testing, cardiac MRI, cognitive evaluation, and multimodal brain MRI. USFC, structural connectivity (SC), and functional connectivity (FC) were computed for predefined 10 backbone pathways. Support vector regression and logistic classification assessed predictive performance.

**Results:**

USFC explained 16%–20% more variance in coronary endothelial function, myocardial perfusion reserve, and cognition than SC or FC alone (*p* < 0.05). Connectivity between the left caudate–superior medial orbital gyrus and right calcarine–inferior occipital gyrus emerged as robust predictors of crystallized cognition (*r* = –0.78, *p*_FDR_ < 0.05) and coronary endothelial function (*r* = 0.70, *p*_FDR_ < 0.05), respectively. USFC also best discriminated low versus high coronary blood flow (area under the ROC curve [AUC]: USFC 0.622 vs. SC 0.349 and FC 0.425; *p* < 0.05).

**Conclusions:**

USFC identifies neuro–cardiac pathways linking cerebral efficiency with coronary endothelial function. These results introduce a sensitive biomarker of systemic vulnerability, highlighting occipital and frontostriatal pathways as shared substrates of dysfunction. USFC offers a mechanistic framework for detecting vascular risk across metabolically demanding tissues.

**Trial Registration:**

ClinicalTrials.gov identifier: NCT03876223

## Graphical abstract

The Unified Structural and Functional Connectivity (USFC) “traffic map” reveals neural pathways linking cerebral efficiency to coronary endothelial function in women with INOCA. This novel biomarker outperforms traditional connectivity measures, highlighting occipital and frontostriatal circuits as shared substrates of systemic microvascular vulnerability.

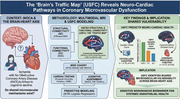

## 1 Introduction

The growing recognition of a brain–heart axis in systemic vascular disease necessitates novel biomarkers that can capture their interconnected pathology, yet how the brain's integrated network architecture predicts cardiac function and associated cognitive decline remains poorly understood (Mejia‐Renteria et al. 2023; Bradley and Berry 2023). A key challenge is mapping how the brain's fixed structural wiring supports its vast functional dynamics—specifically, which anatomical routes are most efficient for communication (Bullmore and Sporns 2009; Vohryzek et al. 2025; Breakspear 2017; Atasoy et al. 2016). To address this, we previously developed the Unified Structural and Functional Connectivity (USFC) model, a novel framework that creates the brain's first integrated “traffic map” by modeling the functional load on each structural segment under an “economical assumption” (Has Silemek et al. 2024). In healthy adults, this model identified a critical midline “backbone” whose efficiency is a superior predictor of cognitive performance than either structural (SC) or functional (FC) connectivity alone (Has Silemek et al. 2024).

Despite this advance, a critical question remains unanswered: can this integrated model reveal clinically meaningful insights in a population where the brain's communication system is under pathological stress? To address this, women with suspected ischemia and no obstructive coronary artery disease (INOCA) due to coronary microvascular dysfunction (CMD) provides a window. This condition, characterized by symptoms of ischemia such as chest pain despite no significant blockages in the major coronary arteries, is linked to coronary and systemic microvascular dysfunction that elevates the risk for both adverse cardiovascular events and cognitive decline (Mejia‐Renteria et al. 2023; Bradley and Berry 2023; Soda et al. 2024). In its potential pathological chain of actions, the brain's SC and FC likely play an important role mediating the impacts of adverse heart conditions on the brain and subsequently cognitive functions. In this study, we applied our framework to participants from the well‐characterized Women's Ischemia Syndrome Evaluation (WISE) (NCT03876223) cohort to bridge the gap between theoretical modeling and clinical application of USFC and test whether the brain's “traffic map” is a sensitive biomarker for the interconnected health of the brain and heart.

We hypothesized that the efficiency of the USFC backbone would not only predict cognitive deficits but would also be directly associated with physiological measures of cardiac function, outperforming conventional SC and FC analyses in both domains. By testing our model in this high‐risk clinical context, this work aims to validate the utility of the USFC “traffic map” as a novel tool for understanding neuro–cardiac vulnerability and to open a new window for the development of integrated biomarkers for both brain and body health.

## 2 Methods

### 2.1 Study Cohort

Thirty‐three women with suspected INOCA due to CMD were consecutively enrolled in the brain ancillary substudy of the WISE study (NCT03876223). Inclusion required symptomatic angina or anginal equivalent, age > 18 years, and no obstructive CAD (< 50% stenosis on invasive coronary angiography), as assessed at Cedars‐Sinai Medical Center. Exclusion criteria included acute coronary syndromes, prior revascularization, primary valvular heart disease, known nonischemic etiologies of chest pain, magnetic resonance imaging (MRI) contraindications (including devices or severe renal impairment), major neurological illness, or estimated life expectancy < 4 years. The study was conducted in accordance with ethical standards established by the Institutional Review Board, and all participants provided informed consent.

### 2.2 Clinical and Cardiac Phenotyping

Comprehensive cardiac phenotyping included invasive coronary function testing to quantify coronary flow reserve (CFR) (Pijls et al. 2002) and symptom burden assessment using the Seattle Angina Questionnaire (SAQ) (Spertus et al. 1995) and Patient‐Reported Outcomes Measurement Information System (PROMIS) (Pilkonis et al. 2011) scales. All participants also underwent a standardized cardiac MRI protocol on a 3T Siemens Vida system to derive indices of left ventricular function, myocardial perfusion reserve index (MPRI), myocardial fibrosis (via late gadolinium enhancement [LGE]), and pre‐contrast T1 time. The protocol included

− Cine SSFP sequences for quantification of left ventricular volumes, mass, and function.
− T1 mapping (MOLLI) for pre‐contrast myocardial tissue characterization.
− First‐pass perfusion imaging (rest and pharmacologic/handgrip stress) for measurement of MPRI, analyzed for transmural, endocardial, and epicardial segments.
− LGE imaging for detection of myocardial fibrosis.
− End‐systolic elastance (Ees) for contractility assessment.
− Early diastolic left ventricular strain rate (circumferential and longitudinal).

### 2.3 Cognitive Assessment

Cognitive performance was assessed using the Montreal Cognitive Assessment (MoCA) (Nasreddine et al. 2005) and the NIH Toolbox Cognition Battery (NIHTB‐CB) (Gershon et al. 2013). The MoCA consists of 30 items (30 points), takes 10–15 min to administer, and assesses core cognitive domains including attention, executive functions, language, memory, and visuospatial skills. The NIHTB‐CB, which takes approximately 45 min to administer, assesses attention, processing speed, executive function, memory, and language. Specific NIHTB‐CB subtests included Picture Vocabulary Test and Oral Reading Recognition Test measuring language functions, List Sorting Working Memory Test measuring working memory, Dimensional Change Card Sorting Test and Flanker Inhibitory Control and Attention Test measuring attention and executive functions, Pattern Comparison Processing Speed Test and Oral Symbol Digit Test measuring processing speed, Picture Sequencing Memory Test measuring episodic memory, and Rey Auditory Verbal Learning Test measuring verbal learning and immediate memory. Composite scores include the Total Cognition Composite, the Fluid Cognition Composite (comprising the Dimensional Change Card Sort, Flanker Inhibitory Control and Attention, Picture Sequence Memory, List Sorting Working Memory, and Pattern Comparison tests), and the Crystallized Cognition Composite (comprising the Picture Vocabulary and Oral Reading Recognition tests).

Patient‐reported outcomes available for the present analyses included PROMIS Applied Cognition Abilities 6a and PROMIS Applied Cognition General Concerns 8a (Cella et al. 2007; Fries et al. 2014). Broader PROMIS emotional distress, fatigue, or social health domains were not available in the analytic dataset and therefore were not included as covariates.

### 2.4 Brain MRI Acquisition and Connectome Processing

#### 2.4.1 Brain MRI Protocol

All participants underwent multimodal brain MRI using a Siemens VIDA 3T scanner with a 32‐channel head coil. High‐resolution T1‐weighted images were acquired using a sagittal 3D MPRAGE sequence (TR = 2300 ms, TE = 2.98 ms, TI = 900 ms, flip angle = 9°, voxel size = 1.0 × 1.0 × 1.0 mm^3^, field of view [FOV] = 256 mm, GRAPPA acceleration factor = 2, 208 slices, acquisition time = 5:12 min). T2‐weighted FLAIR images were also acquired (TR = 4800 ms, TE = 441 ms, TI = 1650 ms, voxel size = 1.0 × 1.0 × 1.0 mm^3^, FOV = 256 mm, 176 slices, GRAPPA = 2, acquisition time = 6:11 min) for structural assessment. Diffusion‐weighted imaging (DWI) was performed using an axial single‐shot echo planar imaging (EPI) sequence (TR = 9600 ms, TE = 81 ms, voxel size = 2.0 × 2.0 × 2.0 mm^3^, FOV = 232 mm, 64 diffusion‐encoding directions, *b* = 0/1000 s/mm^2^, GRAPPA = 2, 80 slices, acquisition time = 10:54 min). Resting‐state functional MRI was acquired with a multiband accelerated axial EPI sequence (TR = 607 ms, TE = 32 ms, flip angle = 50°, voxel size = 2.5 × 2.5 × 2.5 mm^3^, FOV = 220 mm, 64 slices, multiband factor = 8, 976 volumes, acquisition time = 10:00 min, eyes open). All MRI data were visually inspected for motion and artifacts prior to preprocessing and connectome construction.

#### 2.4.2 Connectome Construction

Structural and functional connectomes were constructed using established pipelines. T1‐weighted images were processed with FreeSurfer (v6.0) for brain extraction, cortical and subcortical segmentation, and anatomical parcellation (Destrieux et al. 2010). The Automated Anatomical Labeling (AAL) atlas was registered to each subject's native space using linear and nonlinear transformations to ensure accurate region definition for connectome analysis.

For SC, diffusion‐weighted images were preprocessed and modeled using FSL (Behrens et al. 2007) and MRtrix3 (Tournier et al. 2019) as described previously (Has Silemek et al. 2020). The preprocessing pipeline included corrections for head motion and eddy currents, as well as skull‐stripping, to generate fractional anisotropy (FA) maps. We then estimated white matter fiber orientation distributions using constrained spherical deconvolution and performed whole‐brain probabilistic tractography, generating 150,000 streamlines per subject with a minimum length of 20 mm. To quantify SC strength, streamlines were assigned to pairs of AAL atlas regions, and the mean FA along each connecting tract was calculated. Finally, all SC matrices underwent visual inspection for tract coverage and outliers.

Resting‐state FC preprocessing involved several key procedures, including skull stripping using FSL (Behrens et al. 2007), segmentation of white matter, gray matter, and cerebrospinal fluid via FSL FAST, and motion correction with AFNI (Cox 1996). Participants with framewise displacement > 0.3 mm or < 1000 volumes were excluded. Bandpass filtering in the frequency range of 0.01–0.1 Hz was performed using AFNI, and spatial smoothing was applied via a Gaussian kernel with a full width at half‐maximum of 6 mm. Nonlinear registration of rs‐fMRI images to the Montreal Neurological Institute (MNI) atlas was carried out using ANTs (Avants et al. 2011). Following preprocessing, global signal regression was applied to remove the mean gray matter signal. FC was then computed by measuring the correlation between the average signals of each pair of 90 regions in the AAL atlas, retaining only those correlations that survived a threshold of *p* < 0.05 (false discovery rate [FDR] (Benjamini 1995) corrected).

USFC matrices were constructed as described in our prior work (Has Silemek et al. 2024). For each pair of brain regions with significant FC, we identified the most efficient structural pathway by minimizing a cost function—defined as Euclidean distance divided by SC strength—across all connections up to four steps long as follows:

(1) $$EP=\min\left(\sum_{i=1}^{4}\frac{D}{SC}\;\left(node_{i},\;node_{i+1}\right)\right)\;\;$$

where EP denote the most efficient pathway and *D* and SC reflect Euclidean distance and SC between each pair of AAL connection, respectively. The resulting USFC value for any single structural segment is the cumulative functional “traffic” it carries, calculated by summing the FC weights of all efficient paths that travel along that segment.

In the present analysis, the USFC backbone identified in our prior work (Has Silemek et al. 2024) was utilized as a predefined network of interest. Specifically, the 10 most heavily trafficked USFC segments previously established in a normative cohort—comprising key midline connections among the medial frontal cortex, caudate, thalamus, posterior cingulate, and visual cortices—were selected as the backbone atlas. For each participant, SC, FC, and USFC values were extracted for these backbone connections and used as primary features in all predictive and association analyses. This approach enabled direct evaluation of the generalizability and clinical relevance of the canonical USFC backbone to women with suspected INOCA.

### 2.5 Statistical Analysis and Predictive Modeling

All statistical analyses were performed in R (v4.x) (R Core Team 2023) using established packages. For each cognitive and clinical outcome, all 10 USFC backbone connectivity features were ranked by the absolute value of their univariate correlation with the outcome variable. To prevent data leakage and ensure unbiased model evaluation, feature selection (ranking and selection of the top five features) was performed independently within each training fold during cross‐validation, rather than on the full dataset (Ambroise and Mclachlan 2002). Specifically, for each fold, features were ranked using only the training data, and the top five were selected for model fitting. This procedure was repeated for each modality (USFC, FC, SC) and for each fold.

Missing feature data were imputed via median imputation using caret's preProcess (method = “medianImpute”) (Kuhn 2008). Support vector machine (SVM) regression with a radial basis function kernel was implemented via the caret package in R (method = “svmRadial”), with hyperparameters tuned by internal cross‐validation (Kuhn 2008; Meyer et al. 2021). Model training and evaluation were conducted using k‐fold cross‐validation (number of folds = min(10, max(5, floor[*n*/2]))), where *n* is the number of available subjects for each variable (Kohavi 2001). Model performance was quantified by cross‐validated coefficient of determination (*R* ^2^) and root mean squared error (RMSE), with the highest *R* ^2^ and lowest RMSE across tuning parameter values reported for each modality–outcome pair. Paired *t*‐tests were used to compare *R* ^2^ and RMSE values between USFC, FC, and SC models (*p* < 0.05).

Variable importance was extracted using caret's varImp function, with scores scaled to [0, 100] for each model, and aggregated across outcomes for visualization (Kuhn 2008). Univariate Pearson correlations were computed between all backbone connections and outcome variables (Benesty et al. 2009), with multiple comparisons controlled via the Benjamini–Hochberg FDR procedure (*α* = 0.05) (Hochberg 1995). All analyses were performed in R version 4.2.0 or later, using caret (Kuhn 2008), e1071 (Meyer et al. 2021), ggplot2 (Wickham 2016), patchwork (Pedersen 2020), cowplot (Wilke 2020), dplyr (Wickham et al. 2023), and reshape2 (Wickham 2012).

To complement regression analyses, we conducted a binary classification analysis of coronary endothelial function by dividing participants into low and high coronary blood flow (CBF) groups (median split of the best acetylcholine‐induced CBF at 108 µg dose [Ach108 CBF%] using a 50% cut off). For each connection within the USFC backbone, Cliff's *Δ* was computed to quantify group differences; only edges with *Δ* > 0 (indicating higher connectivity in high‐CBF) were retained as predictive features. Logistic regression models were trained separately for USFC, FC, and SC modalities using the identical *Δ* > 0 features. Model performance was evaluated using 1000 bootstrap resamples (70/30 train/test) to estimate area under the ROC curve [AUC] and 95% confidence intervals [CIs]. Paired *t*‐tests compared AUC distributions across modalities.

## 3 Results

### 3.1 Cohort Characteristics

**FIGURE 1:**
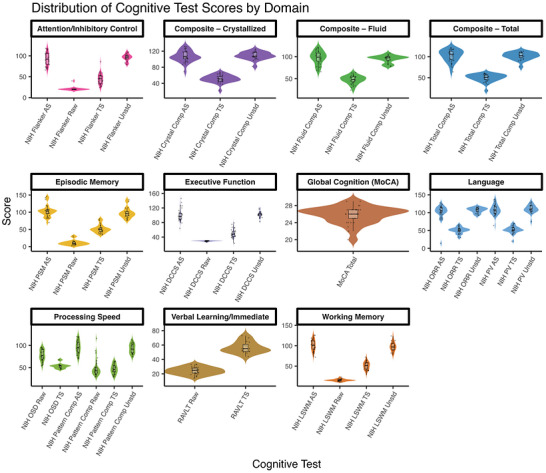
Distribution of cognitive test scores assessed using the Montreal Cognitive Assessment (MoCA) and the National Institutes of Health (NIH) Toolbox—Cognition Battery. This figure presents the distribution of scores from various cognitive tests, including MoCA and NIH Toolbox assessments. Abbreviations: AS, age‐adjusted standard score; MoCA Total, MoCA total raw score; NIH Crystal Comp, NIH Toolbox Cognition Crystallized Composite; NIH DCCS, NIH Toolbox Dimensional Change Card Sort Test; NIH Flanker, NIH Toolbox Flanker Inhibitory Control and Attention Test; NIH Fluid Comp, NIH Toolbox Cognition Fluid Composite; NIH LSWM, NIH Toolbox List Sorting Working Memory Test; NIH ORR, NIH Toolbox Oral Reading Recognition Test; NIH OSD, NIH Toolbox Oral Symbol Digit Test; NIH Pattern Comp, NIH Toolbox Pattern Comparison Processing Speed Test; NIH PSM, NIH Toolbox Picture Sequence Memory Test; NIH PV, NIH Toolbox Picture Vocabulary Test; NIH Total Comp, NIH Toolbox Cognition Total Composite; RAVLT, Rey Auditory Verbal Learning Test; Raw, raw score; TS, age‐ and education‐adjusted *T*‐score; Unstd, unstandardized score (uncorrected for age/education).

The study cohort consisted of 33 women with suspected INOCA (median age 55) from the WISE study, for whom comprehensive cognitive and cardiac data were acquired. Cognitive performance and cardiac function varied across the cohort, as detailed in Table 1 and Figure 1.

**TABLE 1:** Demographics and cardiac outcomes obtained via coronary function testing (CFT), Seattle Angina Questionnaire (SQA), Patient‐Reported Outcomes Measurement Information System (PROMIS), and cardiac magnetic resonance imaging (CMRI) measures in women with suspected ischemia but no obstructive coronary artery disease (INOCA). Abbreviation: MPRI, myocardial perfusion reserve index.

	Women with suspected INOCA due to CMD median (IQR)
Age (years)	55 (17)
Coronary functioning test	
Coronary blood flow (%) (108 mcg)	48.1 (124.7)
Coronary blood flow	25.09 (61.04)
Nitroglycerin diameter response (%)	16.2 (11.7)
Coronary flow reserve	2.9 (1.0)
Systolic blood pressure (mmHg)	121 (24)
Diastolic blood pressure (mmHg)	69 (15)
Seattle Angina Questionnaire	
Seattle Angina Questionnaire 7	52.1 (30.96)
Anginal stability score	50 (0)
Patient‐Reported Outcomes Measurement Information System	
Applied Cognition—Abilities from 6 questions	49.9 (6.6)
Cardiac magnetic resonance imaging	
End systolic elastance (mmHg/mL)	2.51 (1.01)
Left ventricular myocardial mass to volume ratio (g/mL)	0.69 (0.18)
Average pre‐contrast T1 time across the entire left ventricular myocardium	1235.9 (37.5)
Transmural MPRI during pharmacologic	1.16 (0.19)
Epicardial MPRI during pharmacologic stress	1.77 (0.45)
Endocardial MPRI during pharmacologic stress	1.48 (0.32)

### 3.2 USFC Backbone Architecture

**FIGURE 2:**
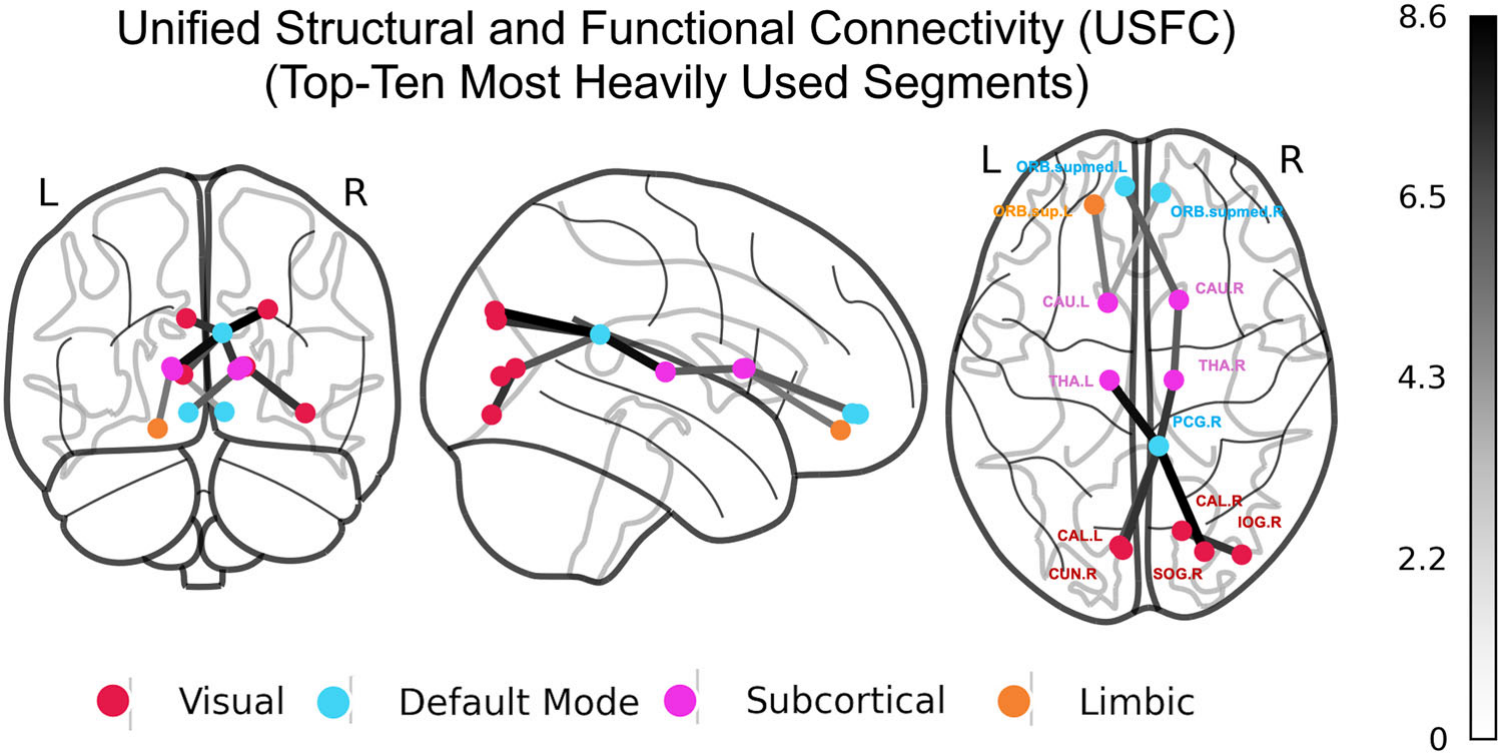
Top 10 most heavily used segments of an USFC “traffic map” in WISE. Node colors indicate the relevant network defined by the Yeo Atlas. Abbreviations: CAL, calcarine cortex; CAU, caudate; CUN, cuneus; IOG, inferior occipital gyrus; L, left; ORBsup, orbital part of the superior frontal gyrus; ORBsupmed, orbital part of the superior medial frontal gyrus; PCG, posterior cingulate gyrus; R, right; SOG, superior occipital gyrus; THA, thalamus. Edges weighted by USFC values are shown in black.

The distribution of USFC values was calculated across the 10 backbone pathways previously defined from healthy subjects in the HCP dataset (Has Silemek et al. 2024). These pathways include midline connections linking the caudate, thalamus, medial frontal cortex, and posterior cingulate to visual cortices, and were evaluated in the WISE cohort (Figure 2).

### 3.3 USFC Yields Superior Cognitive and Cardiovascular Prediction

**FIGURE 3:**
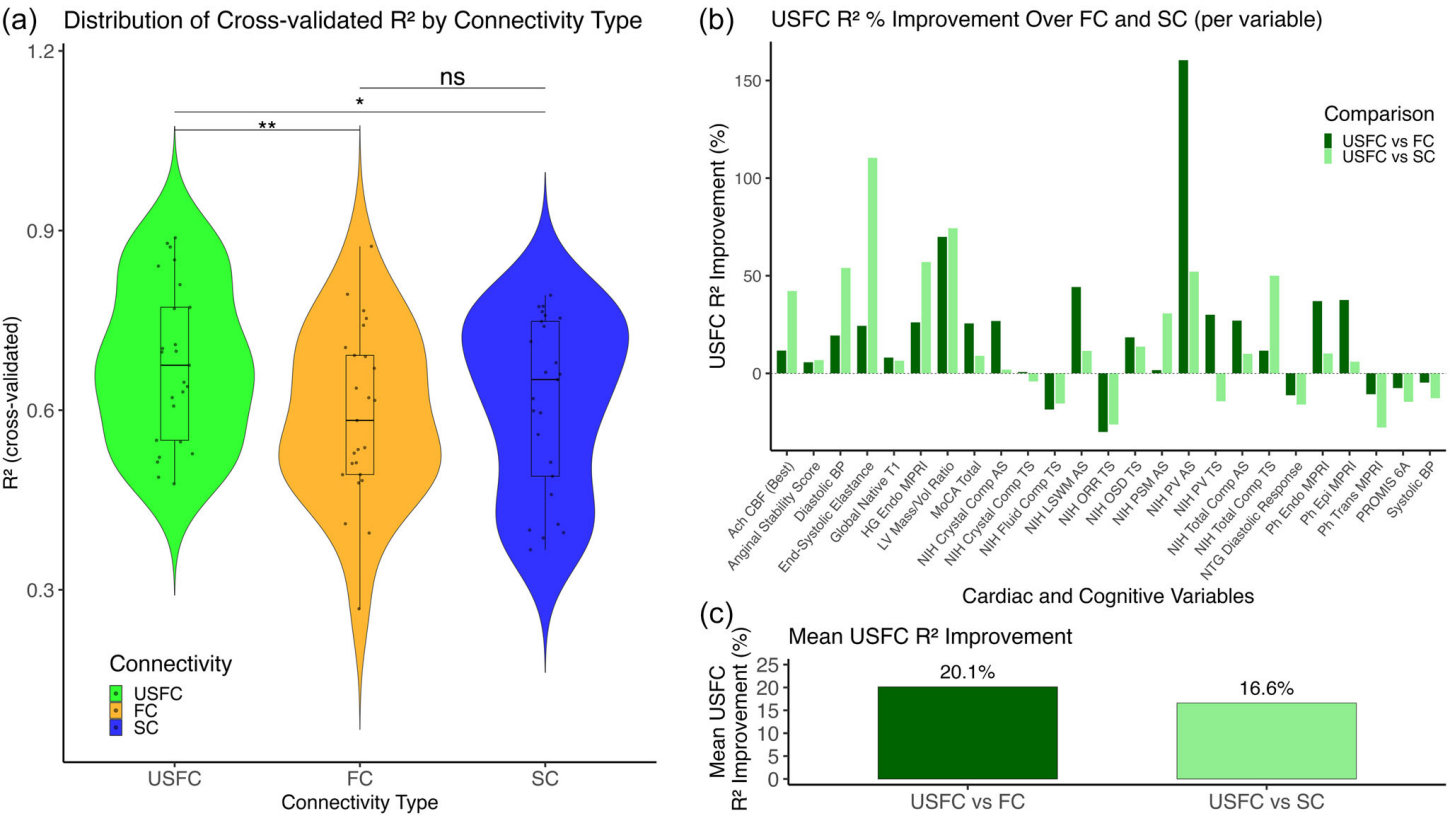
USFC backbone outperforms SC and FC for prediction of cognitive and clinical outcomes. (a) Distribution of cross‐validated *R* ^2^ values for support vector machine regression models predicting cognitive and clinical variables using USFC (green), FC (orange), or SC (blue) backbone connectivity features. Each point reflects a separate outcome variable; boxplots indicate median and interquartile range, with whiskers extending to 1.5 × IQR. USFC models yielded significantly higher predictive accuracy than FC (**p < 0.01) and SC (*p < 0.05) (paired *t*‐test). (b) Percent improvement in *R* ^2^ for USFC‐based models relative to FC and SC models across all outcome variables. Variables represent cognitive test outcomes and cardiac measurements. (c) Mean percent improvement in *R* ^2^ for USFC models compared to FC (20.1%) and SC (16.6%) models, demonstrating the robust predictive advantage of the USFC backbone. Abbreviations: Ach CBF (best), best acetylcholine‐induced coronary blood flow (CBF) at 108 µg dose; AS, age‐adjusted standard score; BP, blood pressure; Endo, endocardial; Epi, epicardial; FC, functional connectivity; HG, Handgrip test; IQR, interquartile range; LV Mass/Vol Ratio, left ventricular mass‐to‐volume ratio; MoCA Total, Montreal Cognitive Assessment total raw score; MPRI, myocardial perfusion reserve index; NIH Crystal Comp, NIH Toolbox Cognition Crystallized Composite; NIH Fluid Comp, NIH Toolbox Cognition Fluid Composite; NIH LSWM, NIH Toolbox List Sorting Working Memory Test; NIH ORR, NIH Toolbox Oral Reading Recognition Test; NIH OSD, NIH Toolbox Oral Symbol Digit Test; NIH PSM, NIH Toolbox Picture Sequence Memory Test; NIH PV, NIH Toolbox Picture Vocabulary Test; NIH Total Comp, NIH Toolbox Cognition Total Composite; ns, not significant; Ph, pharmacological stress; PROMIS 6A, Applied Cognition—Abilities (6‐item short form) from the Patient‐Reported Outcomes Measurement Information System (PROMIS); *R* ^2^, coefficient of determination; Raw, raw score; SC, structural connectivity; SVM, support vector machine; Trans, transmural; TS, age‐ and education‐adjusted *T*‐score; Unstd, unstandardized score (uncorrected for age/education); USFC, Unified Structural and Functional Connectivity.

Using these preselected backbone connections, we tested our primary hypothesis that its integrated structural–functional properties would hold superior predictive power for clinical measures. SVM regression analyses revealed that models leveraging USFC backbone connectivity features achieved significantly greater predictive accuracy for both cognitive and cardiac measures compared to those based on SC or FC alone. As shown in Figure 3a, the distribution of cross‐validated *R* ^2^ values for USFC models was markedly higher than for FC (*p* = 0.006) and SC (*p* = 0.042) models, as determined by paired *t*‐tests across outcome variables, with no significant difference observed between FC and SC models (*p* = 0.445). The predictive advantage of USFC was consistently observed across individual outcomes to correspond to cardiac measurements (Figure 3b). On average, USFC models demonstrated a 20.1% increase in predictive variance explained relative to FC models and a 16.6% increase relative to SC models (Figure 3c).

### 3.4 Pathway‐Specific Associations With Cognition and Cardiac Measures

**FIGURE 4:**
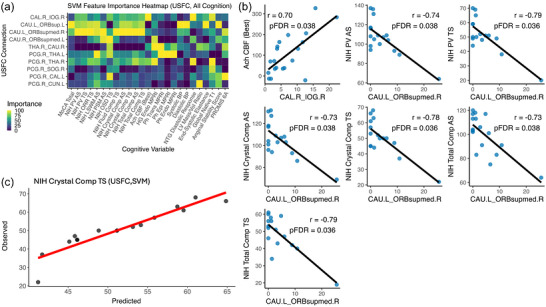
Key USFC backbone connections drive prediction of cognitive and cardiac outcomes. (a) SVM variable importance heatmap illustrating the contribution of each USFC backbone connection (rows) to predictive models of individual cognitive and cardiac variables (columns). Higher values indicate greater relative importance for a given outcome. (b) Representative scatterplots demonstrating significant associations (after FDR correction) between individual USFC backbone connection strengths and cognitive or cardiac outcomes. Shown are examples with robust negative or positive correlations; the FDR‐adjusted *p*‐value (*p*FDR) and Pearson's *r* value are indicated in each plot. (c) Observed versus predicted plot for a representative SVM regression model predicting the NIH Toolbox Cognition Composite Score from USFC backbone features, illustrating model accuracy. Abbreviations: Ach CBF (best), best acetylcholine‐induced coronary blood flow (CBF) at 108 µg dose; AS, age‐adjusted standard score; BP, blood pressure; CAL, calcarine cortex; CAU, caudate; Endo, endocardial; Epi, epicardial; FDR, false discovery rate; HG, Handgrip test; IOG, inferior occipital gyrus; LV Mass/Vol Ratio, Left ventricular mass‐to‐volume ratio; MoCA, Montreal Cognitive Assessments; MoCA Total, Montreal Cognitive Assessment total raw score; MPRI, myocardial perfusion reserve index; NIH, National Institutes of Health; NIH Crystal Comp, NIH Toolbox Cognition Crystallized Composite; NIH Fluid Comp, NIH Toolbox Cognition Fluid Composite; NIH LSWM, NIH Toolbox List Sorting Working Memory Test; NIH ORR, NIH Toolbox Oral Reading Recognition Test; NIH OSD, NIH Toolbox Oral Symbol Digit Test; NIH PSM, NIH Toolbox Picture Sequence Memory Test; NIH PV, NIH Toolbox Picture Vocabulary Test; NIH Total Comp, NIH Toolbox Cognition Total Composite; ORBsupmed, superior medial orbital gyrus; PCG, posterior cingulate gyrus; Ph, pharmacological stress; PROMIS 6A, Applied Cognition—Abilities (6‐item short form) from the Patient‐Reported Outcomes Measurement Information System (PROMIS); Raw, raw score; SOG, superior occipital gyrus; SVM, support vector machine; THA, thalamus; Trans, transmural; TS, age‐ and education‐adjusted *T*‐score; Unstd, unstandardized score (uncorrected for age/education); USFC, Unified Structural and Functional Connectivity.

To understand what drove this superior performance, we identified the specific backbone pathways most critical for predicting clinical outcomes. Variable importance analysis identified the left caudate–right superior medial orbital gyrus pathway as the single most predictive feature for crystallized cognition, which encompasses vocabulary performance (e.g., NIHTB Crystallized Cognition Composite, *r* = –0.78, *p*FDR = 0.036; NIHTB Picture Vocabulary *t*‐score, *r* = –0.79, *p*FDR = 0.036) (see Figure 4a,b). In addition, USFC strength within the visual cortex, specifically between the right calcarine and right inferior occipital gyrus, was strongly associated with coronary endothelial function (*r* = 0.70, *p*FDR = 0.038) (Figure 4b). Thalamus–posterior cingulate connectivity emerged as a preferential correlate of myocardial perfusion reserve under pharmacologic stress (Figure 4a).

The SVM model predicting crystallized cognition composite scores demonstrated excellent concordance between observed and predicted values (Figure 4c). Overall, post‐hoc analyses confirmed multiple robust associations between USFC backbone connections and both cognitive and cardiac indices following correction for multiple comparisons.

### 3.5 Validation of USFC‐Specific Predictive Information Using Classification Analysis

To further evaluate the modality‐specific discriminative capacity of USFC‐derived features for cardiac outcomes, we performed a logistic regression classification of low versus high CBF groups (median split of Ach108 CBF% using a 50% cutoff). Feature selection was based on the USFC backbone edges with positive Cliff's delta (*Δ* > 0), indicating stronger connectivity in the high‐CBF group.

**FIGURE 5:**
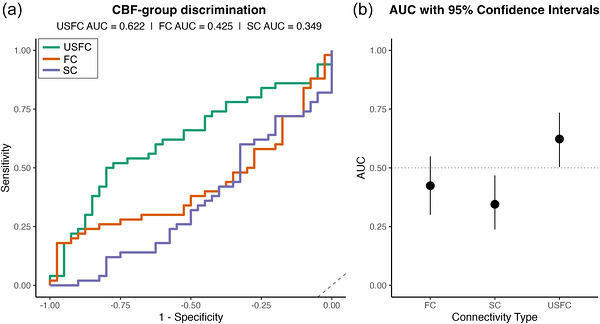
Cross‐modality discrimination of low versus high coronary blood flow (CBF) groups using USFC‐derived features. (a) Receiver operating characteristic (ROC) curves showing model performance for each modality (USFC, FC, SC) using the same five USFC‐derived connectivity edges. The USFC model achieved the highest discriminative accuracy (AUC = 0.622), followed by FC (AUC = 0.425) and SC (AUC = 0.349). (b) Mean AUC values with 95% bootstrap confidence intervals (*n* = 1000 resamples) confirm the superior predictive performance of USFC compared to FC and SC. Results demonstrate that integrated USFC features capture modality‐specific information most relevant to cerebrovascular endothelial function.

The three retained USFC edges were right calcarine—right inferior occipital gyrus (*Δ* = 0.45), right posterior cingulate gyrus—right thalamus (*Δ* = 0.30), and right posterior cingulate gyrus—left cuneus (*Δ* = 0.112). For each modality (USFC, FC, and SC), the same *Δ* > 0 features were used to ensure a fair, feature‐matched comparison. Model performance was evaluated across 1000 bootstrap resamples, with results summarized as mean AUC and 95% CIs (Figure 5). The USFC model achieved the highest discriminative accuracy (AUC = 0.622 [95% CI: 0.50–0.74]), followed by FC (AUC = 0.425 [95% CI: 0.30–0.55]) and SC (AUC = 0.349 [95% CI: 0.23–0.47]). Paired *t*‐tests on the bootstrapped AUC distributions confirmed that USFC significantly outperformed both FC and SC (*p* < 0.05) (Figure 5). These results reinforce that USFC captures unique and generalizable connectivity information most predictive of coronary endothelial function.

## 4 Discussion

This study represents the first application of the USFC model in a clinical population, moving beyond theoretical modeling to uncover potential links between the brain's integrated communication architecture and combined heart–brain health. Based on our “economical assumption,” we demonstrate that the brain's “traffic map” in women with suspected INOCA not only predicts cognitive function but is also robustly associated with cardiac indices. The key finding of this work is the identification of specific, heavily‐trafficked neural pathways whose efficiency is tied to both brain and heart health. This provides compelling new evidence for a unified neuro–cardiac axis of vulnerability in systemic microvascular disease and underscores the superiority of the integrated USFC connectome in revealing these relationships compared to SC or FC measures alone.

Our analysis pinpointed specific segments of the USFC backbone that drive these brain–body associations, each playing a distinct but complementary role. The robust association of the left caudate–right superior medial orbital gyrus pathway with crystallized cognition is of particular note. As a core component of executive control circuits, the caudate acts as a high‐level “executive integrator,” crucial for planning and learning from experience to guide behavior (Choi et al. 2012; Kim and Hikosaka 2015; O'Rawe and Leung 2022). The efficiency of this frontostriatal traffic route is therefore a direct measure of the brain's capacity for complex cognition, explaining its strong predictive link to the cognitive scores in this cohort. Notably, crystallized cognition (i.e., vocabulary) is considered a more stable cognitive construct when compared to cognitive tasks mediated by fluid cognitive constructs, the latter constructs are influenced by dynamic and context‐dependent neural processes, resulting in higher predictability of tasks mediated by crystallized cognitive constructs as found in this cohort (Barbey 2018; Xu et al. 2023). Interestingly, the association between frontostriatal USFC and cognition was negative, indicating that higher structural–functional traffic in this pathway correlates with lower cognitive performance. This finding aligns with the “neural efficiency” hypothesis, which posits that more efficient neural networks require less activation or connectivity to achieve optimal performance (Neubauer and Fink 2009). In the context of microvascular dysfunction, increased network load on specific backbone pathways may reflect compensatory recruitment or “neural inefficiency,” where the brain requires more “traffic” to maintain cognitive output in the presence of vascular constraints (Cabeza et al. 2018).

The frontostriatal and medial frontal pathways highlighted here should also be interpreted within a broader affective and interoceptive context. Prior work in coronary artery disease has shown that mental stress and angina are associated with neural responses in frontal regions, including inferior frontal cortex, and that stress‐related brain activation patterns may differ by sex (Moazzami et al. 2020; Wittbrodt et al. 2020; Kasher et al. 2019). Moreover, sex‐specific stress‐evoked activation of frontal and posterior cingulate regions has been associated with angina severity and coupled with autonomically mediated vasoconstriction and myocardial ischemia, suggesting that these circuits may function as central mediators of the brain–heart stress response (Moazzami et al. 2020; Wittbrodt et al. 2020; Kasher et al. 2019; Bremner et al. 2023). These observations support the possibility that USFC pathways involving caudate, medial/orbital frontal cortex, thalamus, posterior cingulate, and visual cortex may reflect not only cognitive control and network efficiency, but also stress responsivity, symptom perception, and mood‐related modulation of the brain–heart axis. Because broader mood, anxiety, fatigue, and social‐function measures were not available in the current analytic dataset, we could not determine whether these factors mediate or confound the observed associations. Future larger WISE analyses incorporating PROMIS emotional distress/fatigue domains and stress‐provocation paradigms will be important to dissociate cognitive, affective, and autonomic contributions to USFC‐based neuro–cardiac vulnerability.

Of further significance is the direct brain–heart link revealed by the thalamus–posterior cingulate connection, which was a preferential correlate of myocardial perfusion reserve during stress. The thalamus functions as the brain's central “brain–body relay” for interoception—the sense of the body's internal state—processing and transmitting vital signals like heart rate and blood pressure (Wei et al. 2023; De Falco et al. 2024). The efficiency of this pathway reflects how well the brain can sense and respond to the heart's needs (Dong et al. 2020). An inefficient pathway, as captured by USFC, signifies a potential breakdown in this critical feedback loop, providing a clear mechanistic link to impaired cardiac function under stress.

The classification analysis provides convergent evidence that integrated USFC features capture modality‐specific information most predictive of endothelial function. Importantly, this finding aligns with the direct association observed between CBF and the USFC connectivity between the right calcarine and right inferior occipital gyrus, which exhibited the largest positive effect size among all evaluated connections. This suggests that occipital–frontal coupling may reflect vascular regulatory integrity. Prior studies have shown that regional differences in cerebrovascular endothelial reactivity are particularly evident in the posterior (occipital) circulation, where L‐arginine–induced vasodilation of the posterior cerebral artery reflects localized endothelial function (Pretnar‐Oblak 2014). Furthermore, cerebral endothelial function correlates with systemic vascular health (Perko et al. 2011), supporting a shared endothelial regulatory mechanism across cerebral and peripheral vasculature. Notably, disturbances in cerebral blood flow regulation and vascular stiffness have been implicated in cognitive decline and Alzheimer's disease, linking microvascular dysfunction to early neurodegenerative processes (Tarumi and Zhang 2014; Willie et al. 2014). Together, these results suggest that USFC‐derived connectivity, particularly involving occipital and prefrontal regions, captures physiologically meaningful information about cerebrovascular endothelial integrity and may serve as a sensitive indicator of vascular contributions to brain function.

Intriguingly, USFC strength within the visual cortex was strongly associated with coronary endothelial function. This connection likely acts as a “system health indicator.” The visual cortex is one of the most metabolically demanding regions of the brain, making it highly sensitive to changes in blood flow (Wong‐Riley 2010). Poor endothelial function is a systemic issue affecting microvasculature in both the heart and the brain (Bradley and Berry 2023; De La Riva et al. 2024). Therefore, inefficient communication within the visual cortex is not necessarily causing cardiac dysfunction, but rather serves as a sensitive, noninvasive marker of the same underlying vascular pathology that compromises both systems.

The superiority of USFC in identifying these specific relationships highlights a key methodological point: it is the pattern of use of the structural “road system” that carries the most explanatory power. In a population with suspected microvascular disease, subtle inefficiencies in this system affecting how the brain chooses the optimal structural pathway for specific functional communications—which the USFC “traffic map” is uniquely designed to capture—may precede the overt structural or functional damage detectable by conventional methods.

Although this work provides a new perspective, several limitations should be noted. First, our sample size reflects the logistical rarity of simultaneous multimodal MRI and invasive coronary physiology but necessitates caution regarding generalizability. We mitigated overfitting risks via rigorous nested cross‐validation and feature selection strictly within training folds, yet the cross‐sectional design limits causal inference regarding the priority of microvascular versus neural dysfunction. Second, the observed visual cortex associations likely stem from the occipital lobe's high metabolic demand and blood supply via the posterior circulation, a vascular bed with distinct endothelial vulnerability. Third, autonomic biomarkers were not available in the present WISE brain ancillary cohort. Thus, we could not directly test whether USFC backbone alterations reflect brain–brainstem–heart communication or peri‐vascular sympathetic mechanisms involving norepinephrine, neuropeptide Y, nitric oxide signaling, or other indices of autonomic dysregulation. Future studies incorporating autonomic physiology, neurochemical biomarkers, and brainstem‐resolved imaging will be important to determine whether the USFC “traffic map” captures central regulatory pathways that stratify endothelial and flow‐mediated CMD. Fourth, resting‐state fMRI was acquired with eyes open, a condition known to alter connectivity relative to eyes‐closed acquisition, including increased coupling and stability within visual/occipital and attentional systems (Agcaoglu et al. 2019; Han et al. 2023). Therefore, the occipital USFC associations observed here should be interpreted in the context of the eyes‐open acquisition state and should be compared with eyes‐closed protocols in future studies. Finally, the current USFC model relies on static FC; incorporating dynamic fluctuations may further enhance its sensitivity to the real‐time interplay between neural and cardiac systems.

In summary, this study demonstrates that the integrated USFC “traffic map” framework provides a more comprehensive and clinically meaningful characterization of brain network organization in women at risk for cognitive and cardiac dysfunction. By quantifying the efficiency of backbone pathways, the USFC model advances our understanding of brain–body communication and offers a promising platform for the development of predictive biomarkers and therapeutic targets.

## 5 Conclusions

In conclusion, by applying the USFC framework to a clinical population, we have transitioned from theoretical mapping of the brain's “traffic system” to using it as a potential tool to understand subtle brain connectivity alterations in a special clinical population with jeopardized neuro–cardiac health. The clear and stronger association between the efficiency of the brain's USFC backbone and both cognitive and cardiac measures opens a potential new window for understanding systemic disease. Overall, the USFC model offers a promising platform for developing sensitive biomarkers, not just for brain health, but for integrated brain–heart vulnerability.

### 5.1 Clinical Perspectives

#### 5.1.1 Competency in Medical Knowledge

Women with INOCA often present with a clinical paradox—debilitating angina and cognitive symptoms despite preserved epicardial structure. This study shows that a novel “traffic map” of brain network efficiency, USFC, is strongly associated with invasive measures of coronary endothelial function. These findings suggest that occipital and frontostriatal circuits share a common microvascular vulnerability with the heart, reframing INOCA as a systemic disorder of endothelial integrity rather than an isolated cardiac condition.

#### 5.1.2 Translational Outlook

The superior predictive performance of USFC over conventional connectivity models supports its potential as a sensitive, noninvasive biomarker of systemic microvascular health. Future translation will require validation in larger and longitudinal cohorts, assessment of causal pathways, and standardization of the USFC computational workflow for clinical use. Additional studies should test whether therapies targeting CMD can also restore cerebral network efficiency, potentially reducing vascular cognitive impairment in at‐risk women.

## Author Contributions

**Yibin Xie**: investigation, methodology, writing – review and editing, supervision. **Debiao Li**: investigation, supervision, writing – review and editing. **Janet Wei**: investigation, methodology, writing – review and editing, supervision. **Sarah Kremen**: investigation, writing – review and editing, supervision. **Oana Dumitrascu**: investigation, writing – review and editing. **Wei Gao**: resources, supervision, project administration, writing – review and editing, investigation, conceptualization, funding acquisition. **Pascal Sati**: investigation, writing – review and editing, supervision, project administration, resources. **Mitzi Gonzales**: investigation, writing – review and editing, supervision.

## Funding

This work was supported by contracts from the National Heart, Lung and Blood Institutes nos. N01‐HV‐68161, N01‐HV‐68162, N01‐HV‐68163, N01‐HV‐68164, grants U0164829, U01 HL649141, U01 HL649241, K23 HL105787, K23 HL125941, K23 HL127262, K23HL151867 T32 HL69751, R01 HL090957, 1R03 AG032631, R01 HL146158, R01HL124649, PR150224P1 (CDMRP‐DoD), U54AG065141, U54AG094168, GCRC grant MO1‐RR00425 from the National Center for Research Resources, the National Center for Advancing Translational Sciences grant UL1TR000124, the Edythe L. Broad and the Constance Austin Women's Heart Research Fellowships, Cedars‐Sinai Medical Center, Los Angeles, California, the Barbra Streisand Women's Cardiovascular Research and Education Program, Cedars‐Sinai Medical Center, Los Angeles, the Linda Joy Pollin Women's Heart Health Program, the Erika Glazer Women's Heart Health Project, and the Adelson Family Foundation, Cedars‐Sinai Medical Center, Los Angeles, California.

## Ethics Statement

The study protocol received approval from the Cedars‐Sinai Medical Center Institutional Review Board (IRB). The trial was registered at ClinicalTrials.gov (NCT03876223). All procedures performed in this study involving human participants were conducted in accordance with the ethical standards of the institutional and/or national research committee and with the 1964 Declaration of Helsinki and its later amendments or comparable ethical standards. Amendments to the protocol will be submitted to the CSMC IRB for approval.

## Consent

All participants provided written informed consent to participate in the study after being informed of the objectives, procedures, potential risks, and benefits. Consent was obtained in accordance with the requirements of the Cedars‐Sinai Medical Center Institutional Review Board and the participating institutions. The personal information of these individuals was collected through hard copy case report forms and electronic capture in REDCap, and stored securely on Cedars‐Sinai Medical Center's network via VPN, in compliance with federal HIPAA laws.

## Conflicts of Interest

C. Noel Bairey Merz serves as Board of Director for iRhythm. The other authors declare no conflicts of interest.

## Data Availability

The datasets used and/or analyzed during the current study are available from the corresponding author on reasonable request. A custom MATLAB code for construction of Unified Structural and Functional Connectivity is available online (Has Silemek 2024) at: https://github.com/ArzuHas/USFC.git.
